# Hemadsorption with CytoSorb in Infants with Sepsis: Non-Systematic Review of Cases

**DOI:** 10.3390/jcm13226808

**Published:** 2024-11-13

**Authors:** Aruzhan Borankulova, Vitaliy Sazonov

**Affiliations:** 1 Department of Medicine, School of Medicine, Nazarbayev University, Astana Z05K4F4, Kazakhstan; aruzhan.borankulova@nu.edu.kz; 2 Department of Surgery, School of Medicine, Nazarbayev University, Astana Z05K4F4, Kazakhstan; 3 Pediatric Anesthesiology and Intensive Care Unit, National Research Center for Maternal and Child Health, University Medical Center, Astana Z05K4F4, Kazakhstan

**Keywords:** sepsis, neonate sepsis, pediatrics, CytoSorb, hemadsorption

## Abstract

Sepsis is a severe and potentially life-threatening condition that occurs when the body’s response to an infection damages its own tissues and organs. It can lead to organ failure and death if not treated. Cytosorb is a promising medical device for hemadsorption in pediatric septic patients (under 18 years old). As many studies conclude, Cytosorb results in efficient hemodynamics stabilization. This paper is a nonsystematic review of cases. PubMed-, Google Scholar-, and Scopus-indexed journals were used to collect papers for the research. Overall, 11 pediatric cases (six journal articles) were collected. Reductions in interleukin (IL)-6 and IL-10 levels after hemoperfusion with CytoSorb suggest a potential benefit in modulating the inflammatory response in pediatric patients. Moreover, other septic shock indicators such as C-reactive protein, lactate, procalcitonin, ALT, and AST were also significantly reduced in surviving patients within the first few hours of hemadsorption with CytoSorb. The use of CytoSorb seems to be efficient in managing different sepsis-related conditions, even in neonatal and infant populations, as a valuable supplementary tool. However, overcoming the obstacles associated with the age and weight of pediatric patients might necessitate the creation of CytoSorb devices tailored specifically to their needs.

## 1. Introduction

Sepsis is one of the leading causes of hospital death worldwide and has been recognized as the classic medical disorder of the 20th century [1]. According to Rudd et al. sepsis accounts for 48.9 million cases and 11 million sepsis-related deaths worldwide [2]. In 2017, there were 20 million cases and 2.9 million deaths from sepsis in children under the age of 5 years, which is almost half of all global cases [2]. Sepsis is a severe and potentially life-threatening condition that occurs when the body’s response to an infection damages its own tissues and organs [3]. It can lead to organ failure and death if not treated. Sepsis can be caused by various types of infections, such as bacterial, viral, or fungal infections, and other complications. Regarding neonatal and pediatric sepsis, the cause of sepsis can be attributed to internal utero infection, acquisition of parental or maternal flora, and postnatal acquirement from the surrounding environment or hospital [4]. 

One of the distinguishing features of sepsis is the uncontrolled release of cytokines and various inflammatory substances, leading to persistent low blood pressure, tissue damage, metabolic acidosis, and, ultimately, failure of multiple organs [5]. Sepsis is associated with high levels of inflammatory cytokines such as TNF-α, IL-1β, IL-6, and IL-10 [6]. During sepsis, dysregulated release of inflammatory cytokines such as tumor necrosis factor-alpha (TNF-α), interleukin-1 beta (IL-1β), interleukin-6 (IL-6), and interleukin-10 (IL-10) can contribute to harmful effects [7]. The widespread release of inflammatory mediators can cause inflammation throughout the body and can lead to organ dysfunction [8].

Hemadsorption emerges as a novel approach to blood purification, designed to reduce excessively high levels of both pro-inflammatory and anti-inflammatory mediators that are released during the initial stages of sepsis [9].

In recent studies, it was concluded that CytoSorb treatment may be more suitable when there is a prevailing cytokine “storm” or when organ failure progresses [10]. Moreover, Ankawi et al. concluded that although there is no sufficient evidence to support the use of extracorporeal blood purification methods, adsorption may be a promising tool compared to the other techniques [11]. Furthermore, according to Mehta et al., CytoSorb has received the most extensive research attention within the realm of septic shock, with studies demonstrating improvements in survival rates and reductions in sequential organ failure assessment scores, lactate levels, total leukocyte count, and platelet count, as well as levels of interleukin-6, interleukin-10, and tumor necrosis factor [12].

There are limited studies about Cytosorb implementation for septic shock in neonates and infants; thus, this study would be important for understanding the efficacy of this medical device. It is important to have a deeper knowledge about Cytosorb and its effects, so that it can be further improved and made more appropriate/suitable for children. This study is a non-systematic review of cases and aims to evaluate the effectiveness of Cytosorb for hemodynamic stabilization and hemadsorption in different pediatric septic patients with a body mass equal to or less than 10 kg by reviewing previous studies and case reports based on this topic.

## 2. Materials and Methods

PubMed-, Google Scholar-, and Scopus-indexed journals were used to collect papers for the research from inception to 2023. References identified through searches were subjected to an initial independent review at the abstract level by the main author. Then, full-text articles were gathered if they are potentially relevant. Eligible studies adhered to the following criteria: (1) population: neonates and infant septic patients who underwent septic shock; (2) intervention: extracorporeal hemadsorption using CytoSorb^®^; (3) patient criteria: neonates/pediatric patients with body mass equal or less than 10 kg; (4) outcome: any outcome; (5) study design: case report, review of cases, or retrospective study; (6) general: no conflict of interest identified, nonbiased study. Trials with populations that overlapped those of previously included articles and adult studies were excluded. 

**Figure 1:**
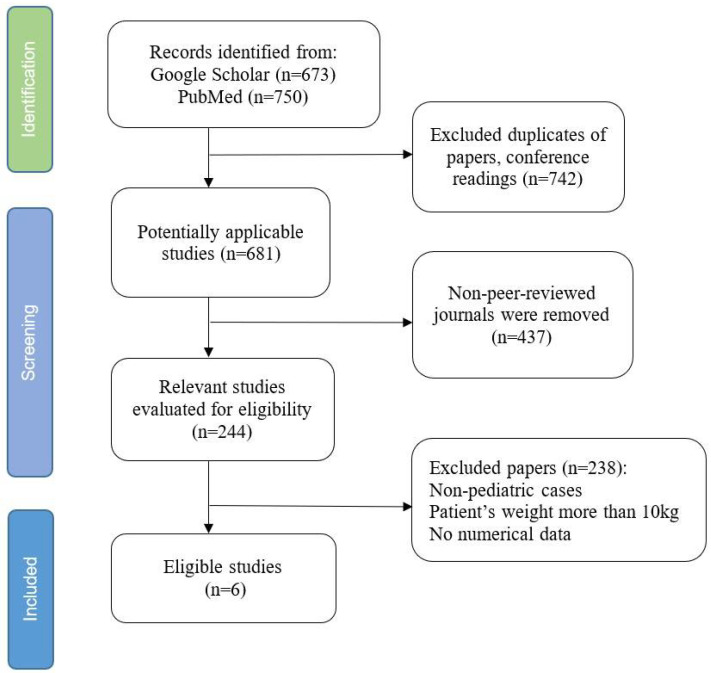
Flowchart of study selection.

Unfortunately, the last article eligible for this study was published in 2021. The articles from Google Scholar, PubMed, and the journals that cited already published papers were reviewed. However, since this study focuses on the patients weighing 10 kg or less, there was a limited amount of papers included for the research. The search and exclusion strategy shown in Figure 1.

Overall, 11 pediatric cases (six journal articles) were collected that follow the criteria of this research. Some articles had more than one case report.

## 3. Results

More than 1400 papers were identified. During the first stages of screening, duplicate articles, conference readings, articles from non-peer reviewed journals, and non-pediatric cases were removed (*n* = 1179). Next, any papers published in non-peer-reviewed journals as well as papers describing cases where the weight of the patient was more than 10 kg were retrieved (*n* = 214). Finally, out of 18 papers, only 6 were chosen as eligible since they follow the criteria stated in the section above. 

In total, six articles were identified that are eligible, five of them are indexed by PubMed, while one other paper is indexed by the Eurasian Scientific Journal Index. However, it is important to note that ESJI may not have the same level of recognition as more widely known indexing services like Web of Science or Scopus. However, these papers include representative and noteworthy data. All cases are summarized briefly in Table 1.

The patients in the studies exhibited various origins of sepsis and septic shock, along with variations in the method, the timing of the initiation, and the frequency of CytoSorb treatment sessions.

This study reviewed 11 cases of septic shock in neonates and infants treated with the CytoSorb hemoadsorption device. Each patient presented with multiple-organ failure and septic shock with various etiology, mainly bacterial infection. Along with the bacterial infection, some patients were presented with complications such as acute kidney failure, cystic fibrosis, myocarditis, and other. However, in this study, the etiology of sepsis was not an important factor. This study focused on evaluation of the effectiveness of CytoSorb based on several criteria, including the reduction in cytokines (IL-6 and IL-10), inflammation parameters (CRP and PCT), lactate levels, duration of pediatric intensive care unit (PICU) stay, levels of aminotransferases, duration of CytoSorb session, and PICU mortality/survival. The cause of septic shock will not be a criterion for inclusion/exclusion. Patients with varying severities of septic shock were enrolled in this study. The changes in the main laboratory data (including their molecular weight) are displayed in Table 2.

## 4. Discussion

Sepsis is the systemic response to an infection. The severity of sepsis depends both on the pathogen and the patient’s reaction to the pathogen [18]. Currently, there exist various blood purification methods with various outcomes, including the removal of cytokines, a reduction in the use of vasopressors, and even a reduction in mortality rates. These techniques include high-volume dialysis, high-cut membranes, filtration-coupled plasma adsorption, and specialized adsorption filters [19].

According to Steurer et al., Cytosorb is a promising medical device for hemosorption in pediatric septic patients (under 18 years of age). As many studies conclude, Cytosorb results in efficient hemodynamic stabilization. Cytosorb has been used to alleviate sepsis in pediatric/neonate patients with different complications such as complex cardiac malformation, acute kidney injury, and others [9]. However, Cytosorb is initially a medical device administered to adults, and its use in sepsis cases is not widely spread [20]. There are many reasons for this: first, most clinical studies for medical devices often focus on adult populations due to ethical and practical considerations. Medical testing in neonatal patients is not common due to the limitations and expensiveness, thus, using such medical devices to treat nonadults is considered unethical. Second, the vascular access required for the use of many devices is challenging in pediatric patients. Third, there are no accurate safety considerations for using Cytosorb for pediatric patient administration. Cytosorb is a hemoadsorption cartridge column featuring hemocompatible porous polymeric beads that eliminate molecules within the 5–60 kDa range, filtering blood from the majority of cytokines and other inflammatory mediators [13]. Cytosorb is a last resort medical device that is used in cases where standard conventional therapy has not shown adequate improvement during the first few hours of signs of septic shock. CytoSorb stands out as the initial extracorporeal cytokine adsorber to gain specific approval within the European Union. With a usage history that exceeds 212,000 treatments to date, CytoSorb is available in 75 countries worldwide [13]. The aim of CytoSorb is to decrease cytokine levels to a point where they are no longer harmful, while preserving the integrity of the immune system. During CytoSorb therapy, blood undergoes purification through adsorption. The adsorber, with a collective surface area exceeding 45,000 square meters, is guaranteed to efficiently reduce elevated cytokine levels over a period of up to 24 h [9]. Primarily targeting hydrophobic (water-insoluble) molecules, it can eliminate substances up to a size of approximately 60 kD, including cytokines. This removal process is contingent on concentration, thus mitigating the risk of complete elimination of physiological mediators [10].

This study is a review of cases related to the treatment of sepsis in infants via hemoadsorption with CytoSorb. In most cases, CytoSorb has been used as a last resort treatment option in case following standard conventional therapy did not result in successful sepsis alleviation. Specifically, focusing on hydrophobic (water-insoluble) molecules, CytoSorb has the ability to remove substances up to a size of around 60 kD, including cytokines. This elimination mechanism is dependent on concentration, thus reducing the likelihood of completely eliminating physiological mediators [10].

One of the criteria for the evaluation of CytoSorb was the level of anti- and pro-inflammatory cytokines in blood before and after application of the CytoSorb device. Septic conditions are characterized by elevated levels of inflammatory cytokines, including IL-6 and IL-10, which are believed to contribute to the development of the condition [21]. Therefore, these cytokines are important to analyze when evaluating the effectiveness of CytoSorb. According to the results of the study, CytoSorb effectively removes blood from cytokines. For example, in Patient 1, the pretreatment IL-6 result was 7821.731 mg/mL, while after treatment IL-6 showed 175.35, which is a 44-fold decrease. Independent of the outcome of the treatment (ICU survival or mortality), IL-6 levels decreased in each patient. As for IL-6, the IL-10 levels for each patient have decreased significantly. In Patient 1, IL-10 before CytoSorb was 2001.29, while after CytoSorb treatment it was 74.34, which is an almost 27-fold decrease in cytokine level. Thus, based on this information, it can be postulated that CytoSorb effectively clears plasma out of pro-inflammatory and anti-inflammatory cytokines IL-6 and IL-10, which is important for septic shock alleviation. According to another neonate study by Christophel-Plathier et al. that has not been included in this study, by decreasing cytokine levels, CytoSorb may have potentially reduced the duration of catecholamine infusion, intubation, and length of stay in the intensive care unit [22]. One of the papers included in this study reported that the median removal ratio for IL-6, IL-10, and TNF-α was 80%, 90%, and 29% [17]. This is a significant result. Adult studies also confirm the effectiveness of CytoSorb in reducing the level of cytokines. For example, Schadler et al. concluded that the CytoSorb device was found to be safe, with observed IL-6 elimination rates ranging from 5% to 18% over the 6 h period [23]. However, there is no need to compare neonate CytoSorb treatment with adult CytoSorb treatment, since it is more complicated for neonates and conclusions should be made only by analyzing neonate septic shock cases.

CytoSorb has also shown its effectiveness in reducing C-reactive protein levels. According to Saparov et al., the CytoSorb therapy session led to a reduction in inflammation markers such as interleukin 6 (IL-6), S100, procalcitonin (PCT), and C-reactive protein (CRP) to levels within normal limits within 12 h [16]. In addition, the levels of transaminases, creatine kinase (CK), and troponin were also normalized. Steurer et al. also reported that combined treatment hemadsorption with CytoSorb resulted in a rapid decrease in CRP, PCT, and IL-6 levels shortly after initiation of therapy [9]. This was accompanied by notable improvements in hemodynamic status, characterized by stabilized blood pressure, increased urine output, and reduced lactate levels in the patient. Furthermore, there was a decrease in the need for platelet replacement and other blood products. According to Cirstoveanou et al., the patient before CytoSorb treatment experienced a severe inflammatory response with CRP of 47.76 U/L; however, after the end of treatment, the patient was discharged from the PICU with mild inflammatory syndrome—the level of CRP was 5.27 mg/dL [15]. Furthermore, Milella et al. also concluded that CytoSorb treatment was linked to a significant reduction in all inflammatory mediators, including CRP [17]. Thus, it can be drawn from these results that CytoSorb is effective in reducing CRP levels in neonate patients with septic shock. However, it does not necessarily mean that CytoSorb filters CRP from the blood, the reason for this will be discussed further in this section.

ALT and AST results are one of the most important factors in the diagnosis of septic shock. According to Schupp et al. the AST/ALT ratio served as a reliable diagnostic tool to identify septic shock and predict 30-day all-cause mortality among patients with sepsis and septic shock [24]. Although only two articles in this study presented ALT/AST results, daily monitoring of ALT and AST, along with regular blood counts, contributes significantly to the effective diagnosis and management of septic cases in neonates. Implementing these diagnostic parameters has the potential to reduce rates of morbidity and mortality among neonates. Both patients, whose data on ALT/AST were available (Patient 5 and Patient 6), experienced relief of liver function and a significant decrease in ALT/AST results. For example, in Patient 5, the ALT before/after CytoSorb treatment was 1883.1 U/L and 87 U/L, respectively, while for AST, the number decreased from 4214.5 U/L to 95 U/L after hemoadsorption via CytoSorb. The same was the case for Patient 8, whose ALT levels decreased from 1118.30 U/L to 271.40 U/L, while AST decreased from 2372.22 U/L to 210.80 U/L [15,16]. Therefore, CytoSorb is effective in decreasing ALT/AST levels in neonates with septic shock.

According to Van Rossum et al. procalcitonin (PCT) has been shown to correlate with the severity of various diseases such as urinary tract infections and sepsis, making it a valuable prognostic marker [25]. As such, procalcitonin serves as a useful additional tool for diagnosing bacterial diseases in neonates and children [26]. Furthermore, Habib et al. also conclude that procalcitonin emerges as a highly beneficial biomarker for the timely diagnosis of neonatal sepsis, showing an impressive diagnostic accuracy of 84.2% [27]. Therefore, PCT helps facilitate early clinical decisions on patient management. The research included in this study showed a significant decrease in procalcitonin levels after the implementation of CytoSorb. For example, PCT levels in Patient 6 decreased from 55.73 ng/mL to 1.59 ng/mL, indicating alleviation of sepsis. Procalcitonin serves as an indicator of sepsis, with elevated levels often observed in individuals with bacterial infections, including sepsis. Monitoring procalcitonin levels can help clinicians diagnose and assess the severity of sepsis, as well as guide treatment decisions such as initiation and duration. CytoSorb has demonstrated effectiveness in reducing procalcitonin levels. By removing inflammatory mediators, including procalcitonin, CytoSorb contributes to modulation of the immune response of the body, which is particularly beneficial in conditions like sepsis where procalcitonin levels are often elevated. This reduction in procalcitonin levels may indicate a positive response to treatment and could potentially be correlated with improved clinical outcomes in patients with sepsis or other inflammatory conditions [28].

Lactate is one of the key indicators of sepsis and is commonly used in clinical practice to assess the severity of the condition and guide treatment decisions [29]. Elevated lactate levels indicate tissue hypoperfusion and are associated with a poor prognosis in septic patients. Lee et al. conclude in their paper that both 6 h lactate levels and 6 h lactate clearance were correlated with 30-day mortality in patients with sepsis and septic shock [30]. Thus, lactate has a prognostic value related to sepsis [31]. A study by Yilmaz et al. also revealed that neonates with elevated lactate levels at the sixth hour after delivery were more likely to receive an early clinical diagnosis of sepsis [32]. Therefore, we should maintain close monitoring of cases exhibiting high lactate levels in the 6th hour postnatal to facilitate the early detection of neonatal sepsis. Those cases that included lactate lab results have discussed that an increase in lactate levels indicates an inadequate response to standard therapy, which implies that hemoadsorption or other blood purification methods are needed [14]. The efficacy of CytoSorb can be evident from the results of Patient 2 (7.05 mmol/mL 5.75 mmol/mL) and Patient 7 (4.6 mmol/mL → 3.0 mmol/mL). A reduction in lactate levels can indicate an improvement in tissue perfusion and oxygenation, which can suggest that the severity of sepsis is alleviated.

In general, out of 11 patients from this study, only 5 have recovered and were discharged from the hospital. This can be explained by the fact that CytoSorb, as mentioned before, in most cases is a last resort treatment option during septic shock. Furthermore, those patients had high anti-inflammatory and pro-inflammatory cytokines; for example, in the study by Milella et al., non-survivors showed pretreatment median levels prior to treatment for IL-6 and IL-10 of 1253.45 pg/mL and 826.9 pg/mL, while survivors had significantly lower levels (IL-6 18.9 pg/mL, IL-10 0.5 pg/mL) [17]. Inflammatory cytokines are pivotal in the development of hyperinflammation and sepsis, with their plasma concentrations correlated with outcomes; specifically, mortality rates tend to be highest when levels of pro-inflammatory and anti-inflammatory cytokines are elevated [7].

In addition, according to Milella et al., non-survivor (three out of four non-survivors described in this study) patients received treatment after a delay of more than 48 h and all succumbed during their stay in intensive care [17]. Research conducted in adults by Kogelmann et al. has indicated that those patients who received therapy within the first 24 h exhibited an ICU mortality rate of 69.2%, notably lower than the expected mortality rate of 92.3%. They came to the conclusion that initiating CytoSorb treatment within the first 24 h of sepsis leads to improved outcomes [5].

It is already known that CytoSorb’s primary focus is hydrophobic (water-insoluble) molecules, allowing the elimination of substances up to approximately 60 kD in size, including cytokines [33]. This means that the device will eliminate anything with a size of 50–60 kD, since it does not have a specificity only to cytokines, toxic agents, and other inflammatory factors. According to Perez et al., due to the potential removal of low-molecular-weight molecules such as prostaglandins by CytoSorb, the dosage increased during treatment [34]. Upon removal of CytoSorb, vancomycin levels substantially rose (reaching 45.4 mg/L), resulting in vancomycin intoxication accompanied by acute kidney injury, which resolved within a few days. Furthermore, Steurer et al. also reported that during one of the septic states (Patient 7 in our study), the requirement for platelet transfusions increased from once to twice daily while undergoing CytoSorb treatment [9]. Despite substitution, their platelet count decreased from 10 G/L before CytoSorb initiation to 8 G/L. Following another transfusion, the platelet count rose to 27 G/L, only to drop back down to 5 G/L within a few hours. Thus, platelet count, absolute neutrophil count, and serum albumin levels should be closely monitored at regular intervals to facilitate prompt and suitable substitution when necessary.

According to Bottari et al., the use of adsorption columns presents an appealing option, as these devices can be integrated with CKRT for the simultaneous management of fluid overload and acute kidney injury (AKI) in Patient 2 [14]. In addition, they play a significant role in the cytokine storm linked to organ damage in septic shock. This combined approach offers a comprehensive strategy for managing multiple aspects of critical illness, potentially improving patient outcomes. Cirstoveanou et al. reported that high-volume hemofiltration (HDF) demonstrated safety and effectiveness in urea from the first day of treatment, despite this, patient bilirubin levels continued to gradually increase over time. However, after combining HDF with CytoSorb, the total bilirubin value decreased from 54 to 14 mg/dL, indicating a substantial improvement in the patient’s condition [15]. Furthermore, there was a rapid drop in aminotransferase levels, signifying a positive response to treatment. Hemodynamically, there was improvement, with a rapid reduction in the need for inotropic support. Additionally, the patient’s ventilation settings improved during CytoSorb treatment, allowing for successful weaning from mechanical ventilation after five days of hemadsorption. Overall, the patient’s general status improved significantly during the course of treatment. Kogelmann et al. reported that treating septic patients with a combination of CytoSorb and CVVHD led to notable stabilization in hemodynamics and decreased vasopressor dosing [5]. Furthermore, Steurer et al. concluded that combined treatment involving VA-ECMO, CVVHDF, and CytoSorb hemadsorption resulted in a rapid decrease in CRP, PCT, and IL-6 levels shortly after therapy initiation (Patient 7) [9]. This was accompanied by a significant improvement in hemodynamic status, evident from stabilized blood pressure, increased urine output, and reduced lactate levels in the patient. Furthermore, there was a decreased need for platelet replacement and other blood products. Thus, CytoSorb can be effectively combined with other blood purification techniques. This combination therapy approach allows for the complete management of patients with conditions such as sepsis and organ failure, targeting multiple aspects of the pathophysiology, including the removal of cytokines, the clearance of toxins, and the optimization of fluid balance, thus potentially improving patient outcomes.

One of the most important factors is the molecular weight of molecules and inflammatory mediators. It is known that Cytosorb is able to remove molecules with molecular weight up to 60 kDa [10]. As can be seen from Table 2, CytoSorb is able to adsorb every molecule besides C-reactive protein (CRP). If CRP has a molecular weight of 110 kDa and CytoSorb can only remove substances up to approximately 60 kDa, then CytoSorb may not be able to directly remove CRP from the bloodstream. CytoSorb primarily targets molecules with a molecular weight of up to 60 kDa, including cytokines and other inflammatory mediators. However, it is important to note that CytoSorb’s mechanism of action involves the removal of a broad range of inflammatory mediators, not just specific molecules based on their molecular weight. While CRP may not be directly removed by CytoSorb, the reduction in other inflammatory mediators may indirectly lead to a decrease in CRP levels over time as the inflammatory response is modulated. Additionally, the effectiveness of CytoSorb in clinical settings may vary depending on factors such as the patient’s condition, the specific inflammatory mediators present, and the overall treatment strategy. Therefore, it is essential to consider the broader context of CytoSorb’s therapeutic effects when evaluating its potential impact on CRP levels.

Especially in the case of neonate septic shock, the management of sepsis can be very complicated. CytoSorb is a hemoadsorption device which is originally used for adult sepsis management. Thus, existing safety profile data and application recommendations cannot be readily extended to pediatric patients. The vascular access required for the use of many devices, including CytoSorb, is challenging in pediatric patients. For example, in some cases, two single-lumen catheters placed in different veins were used instead of one double-lumen catheter.

Another consideration is the blood flow rate. The priming volume of the circuit and CytoSorb could be about 270 mL, which constitutes a big portion of neonate blood volume, this range may not be feasible for low-weight neonate patients. In most cases, the authors of the studies included in this review indicated that the primary filling of the circuit, in addition to normal saline, was albumin or blood [14,16,17].

Moreover, there is a lack of available data on the safety and effectiveness of using lower flow rates. Consequently, some studies decided to maintain blood flow rates within the range of 100–150 mL/min due to a scarcity of recommendations specific to pediatric patients. Other studies have reported blood flow rates as low as 40 mL/min without observing negative effects [15]. According to Steurer et al., the blood flow rate being too high can result in severe hypotension or hemodilution. Each patient should be watched carefully [9].

This study has several limitations. Besides the discussion of the results of this research, it is worth mentioning that it was important to include not only case reports but also retrospective studies. This can be explained by the fact that case reports usually include successful outcomes, while retrospective studies can include both successful treatment outcomes and mortality cases. Thus, it can be said that retrospective studies are less biased. The outcomes/conclusion of this study cannot be suitable for each neonate septic shock case and the results can be controversial since, as it was mentioned before, CytoSorb has been used as a last resort treatment if following sepsis protocol did not alleviate patient’s condition. In practice, it is important to choose the right treatment strategy on time; thus, it can be assumed that if CytoSorb was implemented earlier there would be less morbidity and mortality. A recent meta-analysis by Becker et al. found no convincing evidence that CytoSorb has a significant impact on mortality. They also conclude that future efforts should focus on identifying appropriate candidates, such as those with exceptionally high cytokine levels, and determining the optimal timing for initiating therapy in these conditions [33]. This is generally supported by our study.

Focusing on the neonate and infant septic shock cases described in this research, almost in all cases, sepsis etiology was related to bacteria, except for two cases with fungal infection (particularly Candida). If most patients with bacterial infection survived, out of six non-survivors, two of them had fungal infection. Based on our limitations, we envision the development of further studies. As more papers are published on hemoadsorption in children, we will have more statistically reliable data. It is likely that as cartridge volume decreases, hemoadsorption will become more widely used. It is also important to note that there is a need for studies evaluating the long-term effects on recovery and quality of life.

## 5. Conclusions

Using CytoSorb in neonates and infants poses several challenges, primarily due to their unique physiological characteristics and size. For example, neonates have much smaller blood volumes compared to adults, making it challenging to safely implement extracorporeal therapies like CytoSorb without risking significant hemodynamic instability or hemodilution. Second, securing adequate vascular access for neonates can be challenging due to their small veins. Inserting the necessary cannulas for CytoSorb treatment can require specialized expertise and carry a higher risk of complications such as thrombosis or infection. Third, neonates have different metabolic rates and drug clearance mechanisms compared to adults, necessitating careful consideration of dosing regimens and potential drug interactions when using CytoSorb. Next, CytoSorb is primarily designed for use in adults, so adapting device and therapy protocols to meet the unique needs of neonates requires additional research, development, and validation. Moreover, we should have a clear understanding that hemoadsorption is an adjuvant method and should be performed together with the main protocol (antibiotic therapy, infusion, inotropes, etc.). Finally, there are limited safety and efficacy data for the use of CytoSorb in neonates. Cytosorb may not address all the issues, but it could serve as a valuable supplementary tool. Further research and clinical trials are needed to establish the optimal use, safety profile, and potential benefits of CytoSorb in this population.

## Figures and Tables

**Table 1:** Studies included in the research and characteristics.

ID	Study	Gender, Patient’s Weight (kg)	Cause of Sepsis	Time of First Cytosorb Implementation	Cycles of Cytosorb Therapy	Combination with Other Devices	PICU Days	Sepsis Indicators Each Study Focused on	Outcome of the Therapy
1	Bottari et al., 2020 [13]	M, 10 kg	Klebsiella pneumoniae Pseudomonas aeruginosa	Within 24 h of septic shock signs	4 + 3 (24 h each)	ECMO, CRRT	>28	IL-6, IL-10, TNF-alpha	Discharged from PICU after 60 days
2	Bottari et al., 2021 [14]	F, 9 kg	Klebsiella pneumoniae	Within 24 h of septic shock signs	3 (24 h each)	CRRT	22	IL-6, IL-10, lactate	In-hospital mortality
3	Bottari et al., 2021 [14]	F, 3.130	Serratia marcescens	Within 24 h of septic shock signs	1 (24 h)	VA-ECMO	16	IL-6, IL-10, lactate	In-hospital mortality
4	Bottari et al., 2021 [14]	M, 3.600	Serratia marcescens Pseudomonas aeruginosa	Within 24 h of septic shock signs	2 (24 h each)	VA-ECMO	43	IL-6, IL-10, lactate	In-hospital mortality
5	Cirstoveanu et al., 2017 [15]	M, 9 kg	Escherichia coli	Within 24 h of septic shock signs	49 h: 1 cycle	CRRT HDF	34	Bilirubin, ALT, AST, CRP	Discharged home after 34 days
6	Saparov et al., 2019 [16]	M, 5.6 kg	Burkholderia cepacia + Staphylococcus aureus MRSA + Candida albicans	Within 24 h of septic shock signs	36 h: 1 cycle	CVVHD	/	IL-6, S100, ALT, AST, procalcitonin, CRP, leucocytes, troponin	Discharged home
7	Steurer et al., 2021 [9]	M, 7 kg	Enterobacter cloacae complex; Staphylococcus epidermidis.	Within 24 h of septic shock signs	40 h: 1 cycle	VA-ECMO, CVVHDF	28	IL-6, lactate, CRP, procalcitonin	Discharged from PICU after 28 days
8	Milella et al., 2019 [17]	M, 3.5 kg	Candida parapsilosis	After 24–48 h	19 h: 1 cycle	ECMO, CRRT	38	IL-6, IL-10, procalcitonin, CRP, TNF-alpha	In-hospital mortality after 38 days on ICU from cardiac failure
9	Milella et al., 2019 [17]	F, 10 kg	Escherichia coli O157	After 24–48 h	36 h: 2 cycles	ECMO, CRRT	4	IL-6, IL-10, procalcitonin, CRP, TNF-alpha	In-hospital mortality after 4 days on ICU
10	Milella et al., 2019 [17]	F, 8 kg	None	After 24–48 h	6 h: 1 cycle	ECMO	7	IL-6, IL-10, procalcitonin, CRP, TNF-alpha	Discharged home
11	Milella et al., 2019 [17]	F, 4.8 kg	Proteus mirabilis, Candida parapsilosis	After 24–48 h	52 h: 2 cycles	ECMO, CRRT	30	IL-6, IL-10, procalcitonin, CRP, TNF-alpha	In-hospital mortality after 30 days

(VA)ECMO—(Venoarterial) ExtraCorporeal Membrane Oxygenation, CRRT—Continuous Renal Replacement Therapy, CVVHD (F)—Continuous Venovenous Hemodiafiltration, HDF—High-Volume Hemodiafiltration, IL—interleukin, CRP—C-reactive protein, TNF—tumor necrosis alpha, ALT—Alanine aminotransferase, AST—Aspartate transferase.

**Table 2:** Changes in the main laboratory data (including their molecular weight).

	IL-6 (23 kDa)		IL-10 (18 kDa)		CRP (114 kDa)		PCT (13 kDa)		ALT/AST		Lactate	
Cases ID	Before (mg/mL)	After (mg/mL)	Before (mg/mL)	After (mg/mL)	Before (mg/mL)	After (mg/mL)	Before (ng/mL)	After (ng/mL)	Before (IU/L)	After (IU/L)	Before (mmol/L)	After (mmol/L)
1	7821.731	175.35	2001.29	74.34								
2–4	1801.27 *	203.175 *	178.78 *	22.68 *							7.05 *	5.75 *
5					47.76	5.27			1883.1/4214.5	87/95		
6	77.60	47.35			336.19	93.18	55.73	1.59	1118.30/ 2372.22	271.40/210.80		
7	640	77			42	0.5	41	1.8			4.6	3.0
8	1253.45 **	997.67 **	826.9 **	112.87 **	152.6 **	104.7 ***	18.2 **	12.3 **				
9	18.9 ***	9.8 ***	0.5 ***	48.8 ***	148.9 ***	9.5 ***	5.5 ***	1.0 ***				
10–11	1253.45 **	997.67 **	826.9 **	112.87 **	152.6 **	104.7 ***	18.2 **	12.3 **				

Before and after Cytosorb hemoperfusion procedure. *—median result for 8 patients, Bottari et al. (2021) [14] **—median result for non-survivor patients, Milella et al. (2019) [17]. ***—median result for survivor patients, Milella et al. (2019).

## Data Availability

No new data were created or analyzed in this study. Data sharing is not applicable to this article.
